# Twenty four-month follow-up after bullectomy, unilateral and bilateral lung volume reduction surgery: a single-center retrospective analysis of consecutive cases

**DOI:** 10.1186/s40001-024-01879-4

**Published:** 2024-05-10

**Authors:** Slavisa Bascarevic, Maja Ercegovac, Mir Alireza Hoda, Milan Savic, Radomir Vesovic, Vladimir Milenkovic, Marina Moromila, Marko Popovic, Daniela Gompelmann, Petar Madzarevic

**Affiliations:** 1 Clinic for Thoracic Surgery, University Clinical Centre of Serbia, University of Belgrade, Belgrade, Serbia; 2 Department of Thoracic Surgery, Medical University of Vienna, Vienna, Austria; 3 Division of Pulmonology, Department of Internal Medicine II, Medical University of Vienna, Vienna, Austria

**Keywords:** Emphysema, Lung volume reduction surgery, Bullectomy, COPD

## Abstract

**Purpose:**

While pharmacologic therapy remains the cornerstone of lung emphysema treatment, surgery is an additional therapeutic option in selected patient groups with advanced emphysema. The aim of lung volume reduction surgery (LVRS) is to improve lung function, exercise capacity, quality of life and survival. We sought to determine the therapeutic value of surgical resection in specific patients with lung emphysema.

**Patients and methods:**

A retrospective study was performed consisting of 58 patients with lung emphysema who underwent surgical intervention over a 10-year period and were followed for 2 years postoperatively. The clinical characteristics recorded were FEV_1_ (forced expiratory volume in 1 s), the 6-min walk test (6-MWT), the Modified Medical Research Council (mMRC), body mass index (BMI) and quality of life prior to and 6, 12 and 24 months after surgical intervention. Moreover, all peri- and post-operative complications were noted.

**Results:**

Out of 58 emphysema patients (72% male, FEV_1_ (L) 2.21 ± 0.17, RV (L) 3.39 ± 0.55), 19 underwent surgical bullectomy, 31 unilateral LVRS and 8 sequential bilateral LVRS. Six months after surgery, there was a statistically significant improvement in FEV_1_, RV, TLC, 6-MWT and mMRC. Over a period of 12 to 24 months postoperatively, clinical benefit gradually declines most likely due to COPD progression but patients still experienced a significant improvement in FEV_1_. The most common postoperative complications were persistent air leakage (> 7 days), arrhythmia and subcutaneous emphysema in 60%, 51.6% and 22.4%, respectively. No deaths were observed after surgical intervention.

**Conclusion:**

In a selected patient population, surgery led to significant improvement of lung function parameters, exercise capacity and quality of life. Over a period of 12 to 24 months postoperatively, clinical benefit gradually decreased most likely due to COPD progression.

## Introduction

Chronic obstructive pulmonary disease (COPD) is a heterogeneous disease entity that is linked to multiple comorbidities and systemic manifestations. Pulmonary emphysema, a phenotype of COPD is characterized by hyperinflation leading to breathlessness and a reduced quality of life. COPD is currently the fourth leading cause of death in the adult population worldwide [1].

Pharmacologic therapy is the cornerstone of lung emphysema treatment, while surgery remains one of the therapeutic options of choice. Bullectomy and lung volume reduction surgery (LVRS) aim to reduce hyperinflation and thus lead to optimized lung function, exercise capacity, and survival. Several trials have demonstrated that bullectomy may lead to transient alleviation of dyspnea and certain indices of respiratory function that dissipate over time [2, 3]. However, poor results post-bullectomy have been observed over time in patients with advanced emphysema in unresected segments of lung parenchyma (stage III according to the De Vries and Wolf classification) [4, 5].

Lung volume reduction surgery (LVRS) has been accepted as the therapeutic modality of choice for patients in the terminal phase of lung emphysema. To date, the National Emphysema Treatment Trial (NETT) published in 2003, is the largest randomized controlled trial related to LVRS; this study compared 608 patients who underwent LVRS to 610 patients who received medical treatment [6]. Overall, lung volume reduction surgery increased the chance of improved exercise capacity, but did not confer a survival advantage over medical therapy. However, subgroup analysis showed that patients with predominantly upper-lobe emphysema and low exercise capacity in particular experienced improvements in exercise capacity and survival compared to those in the medical therapy group. In contrast, in patients with a forced expiratory volume in 1 s (FEV_1_) ≤ 20%, a transfer factor for carbon monoxide (TLCO) ≤ 20% and a homogeneous emphysema distribution, increased mortality was observed after LVRS. These results demonstrated that LVRS is beneficial after precise patient selection.

In this study, we sought to determine if surgery can be performed safely and lead to improved outcomes in an institutional cohort of consecutive patients with advanced emphysema.

In the era of bronchoscopic LVRS some centers have nearly abandoned the use of surgical LVRS. This paper sought to determine the continued utility, safety and outcomes of surgical LVRS in an institutional cohort of consecutive patients with advanced emphysema who were active smokers or past smokers and belonged to a specific geographic area of Europe.

## Materials and methods

A retrospective study was performed, that included 58 consecutive patients suffering from lung emphysema, who underwent surgical intervention at our institution between 2007 and 2017. All patients were followed for a minimum of 2 years postoperatively. Written consent was obtained from all study patients and the study was approved by the institutional ethics committee (MFUB/25.02.2016.Nr. 29/II-21).

The primary objective of our study was to evaluate the clinical long-term outcomes following bullectomy and unilateral or bilateral LVRS in patients with advanced emphysema.

### Patient eligibility for surgical intervention

Information obtained from individual patient histories included smoking habits and preexisting comorbidities. All patients of the cohort were either active smokers or ex-smokers and ceased smoking at least 4 weeks prior to surgical intervention. All patients with cardiomyopathy, arterial hypertension and type II non-insulin dependent diabetes mellitus were adequately controlled with medical treatment prior to surgical intervention. Standard laboratory tests and pulmonary function tests were carried out for all patients to preoperatively grade the candidates for resection of lung parenchyma as were: body plethysmography, DLCO, arterial blood gas analysis, echocardiography, 6-min walk test (6MWT), body mass index (BMI), Modified Medical Research Council (mMRC) score and BODE score.

To confirm emphysema and evaluate emphysema distribution, computed tomography (CT) of the chest and ventilation/perfusion scintigraphy were performed.

Patients were referred and evaluated by the thoracic surgical team only if they met the NETT criteria. Indications for surgical intervention and the anatomical region for resection were determined using standardized NETT criteria: FEV_1_ > 20%, DLCO > 20%, evaluation of emphysematous morphology and the degree of heterogeneity (analysis of chest CT and ventilation–perfusion scintigraphy). Patients with homogenous emphysema and alpha-1 antitrypsin deficiency were excluded. All patients underwent preoperative lung rehabilitation. Perioperatively all patients received standard medical care consisting of bronchodilator inhalation therapy and respiratory physical therapy. Patients received antibiotic prophylaxis according to a standardized protocol, and further antibiotic therapy was administered in patients with clinically and bacteriologically confirmed infections. The perioperative management also included routine DVT prophylaxis and gastroprotective therapy. All patients who underwent either bullectomy, unilateral LVRS or sequential bilateral LVRS met both surgical eligibility criteria and inclusion/exclusion criteria as outlined for this retrospective study.

### Surgical procedure

The patients included in this retrospective analysis were divided into three groups according to the surgical intervention: bullectomy (group 1, *n* = 19), unilateral LVRS (group 2, *n* = 31) and sequential bilateral LVRS (group 3, *n* = 8). Bullectomy is defined as atypical lung resection of localized parenchymal bulla with the planned resection margin between bulla and healthy or potentially affected lung parenchyma. Lung volume reduction surgery (LVRS) is a unilateral non-anatomic lung resection of the most damaged lung segments with a planned volume reduction of 20–30% of lung volume. In the case of extended emphysema including both upper and lower lung lobes (predominantly apical lower lobe segment), LVRS includes atypical resection of both upper lobe and the apical lower lobe segment to achieve a total of 20–30% of lung volume reduction.

All surgical interventions were carried out through a muscle-sparing anterolateral thoracotomy through the 5th intercostal space. The same surgical team performed each of the operative interventions. Training in the operative techniques for surgical emphysema treatment was obtained over a previous 5-year period according to published surgical protocols. In all the patients the lung with the highest degree of loss of healthy tissue was resected first.

Due to previously reported comparable functional results and non-inferior postoperative morbidity and mortality after sequential bilateral LVRS compared to bilateral LVRS, the former procedure was the procedure of choice [7]. Sequential bilateral treatment was carried out through a delayed approach at an interval of 6–12 months. The timing of the surgical intervention on the contralateral side was determined by a combination of factors, such as the postoperative recovery time after initial resection, degree of improvement in lung function after intensive rehabilitation and patient willingness to undergo an additional operation.

The site and degree of resection were determined using existing criteria for surgical lung volume conservation which allowed for an adequate degree of breathing dynamics and function of the remaining lung. Resection was carried out using a linear stapler (United States Surgical Corporation, Norwalk, CT; Ethicon, Inc, Cincinnati, OH) with a length between 50 and 70 mm. Following resection, fibrin glue was applied on the lung suturing stapler line, to reduce the amount of air leakage.

### Follow-up after surgical intervention

In all patients, institutional standards of postoperative care and physical therapy were applied. The evaluation of postoperative lung function parameters FVC, FEV_1_, RV, TLC, BMI, 6MWT result, degree of dyspnea, use of steroid therapy and quality of life was carried out 6 months, 12 months and 24 months postoperatively. In the bilateral LVRS patient group 6-month, 12-month and 24-month follow-up results were recorded after the 2nd surgical intervention.

### Statistical analysis

A statistical methodology was used to analyze the results of the study. In the first stage of statistical analysis of study results, a database for all patients was constructed, and then organized, grouping patient data into categorized tables and graphs of results. From the descriptive statistical parameters, the arithmetic mean (*X*) with measures of variance (standard deviation SD and standard error SE) of the median, mode, and frequency distribution were calculated. Graphical and mathematical procedures were used to test the normality of distribution. To test the statistical hypothesis, in accordance with the type and distribution of variables, Student’s *t* test, Chi-square test, Mann‒Whitney *U* test and Fisher’s test were used. All statistical tests were executed using a significance level of *p* < 0.05. Analyses were performed in IBM SPSS Statistics for Windows, version 26.0 (Armonk, NY: IBM Corp., 2019).

## Results

### Patient characteristics

Out of the 58 emphysema patients (72% male, FEV_1_ (L) 2.21 ± 0.17, RV (L) 3.39 ± 0.55), 19 underwent surgical bullectomy, 31 unilateral LVRS and 8 sequential bilateral LVRS. The clinical characteristics, including lung function parameters, mMRC and BMI of the patients are summarized in Table 1.

**Table 1:** Preoperative clinical characteristics (PCCs)

Clinical parameter	Bullectomy (*n* = 19)				LVRS unilateral (*n* = 31)				LVRS bilateral (*n* = 8)			
	Min.	Max	Mean	SD	Min.	Max	Mean	SD	Min.	Max	Mean	SD
FVC (L)	2.78	4.36	3.75	0.38	2.79	4.12	3.64	0.38	2.32	3.95	3.23	0.44
FVC (%)	68	87	78.05	5.86	63	83	74.55	4.53	58	76	67.88	5.46
FEV_1_ (L)	1.91	2.63	2.21	0.17	1.39	2.69	2.06	0.25	1.65	2.23	1.89	0.20
FEV_1_ (%)	51	63	58.63	4.45	42	63	52.65	5.49	44	61	51.12	4.94
TLC (L)	4.91	7.12	5.60	0.76	4.91	8.24	6.15	0.90	5.32	7.23	6.51	0.71
TLC (%)	112	138	118.5	7.65	106	138	120.6	8.31	118	139	128.7	6.47
RV (L)	2.86	4.91	3.39	0.55	2.63	5.47	3.84	0.66	3.43	4.93	4.39	0.51
RV (%)	126	183	146.1	11.7	131	275	159	31.9	146	215	175.9	22.7
RV/TLC %	0.39	0.59	0.48	0.04	0.41	0.75	0.52	0.08	0.51	0.63	0.59	0.04
RV/TLC	1.17	1.38	1.28	0.06	1.09	2.32	1.40	0.28	1.16	1.84	1.39	0.19
DLCO (L)	5.3	6.7	5.95	0.34	4.9	6.9	6.01	0.51	5.1	6.7	5.75	0.58
DLCO (%)	57	73	66.6	3.8	51	71	63.1	5.4	61	71	65.1	4.0
6MWT (m)	340	436	395	27.2	326	420	367	28.4	320	410	352	30.6
mMRC	1	2	1.95	0.22	2	3	2.06	0.25	2	3	2.38	0.50
BMI (kg/m^2^)	17	28	23.9	3.2	19	32	24.2	3.5	17	29	19.9	3.9

FVC: forced vital capacity; FEV_1_: forced expiratory volume in 1 s; TLC: total lung capacity; RV: residual Volume; DLCO: diffusing lung capacity of the lungs for carbon monoxide; 6MWT (m): 6-min walking test result in meters; mMRC: Modified Medical Research Council Score; BMI: body mass index

Prior to surgical intervention alongside standardized bronchodilator therapy, 42.1% of our patients in the bullectomy group received corticosteroid therapy, 80.6% of those in the unilateral LVRS group and 87.5% of those in the bilateral LVRS group. With a significant frequency the vast majority of our patients who were followed up to 24 months postoperatively (89.5% to 100% of patients, *p* < 0.001), did not use steroid therapy.

Overall, 51.7% of all patients had significant comorbidities: 28 patients (48.3%) had cardiomyopathy (NYHA class I, NYHA class II) preoperatively, 11 patients (18.3%) had arterial hypertension preoperatively and 6 patients (10.3%) patients had type II non-insulin-dependent diabetes mellitus preoperatively.

Patients who underwent LVRS had heterogeneous pulmonary emphysema, while in the bullectomy patient group preoperative CT findings revealed bullous emphysema mostly predominant in the upper lobes (stage III according to the De Vries and Wolf classification). The majority of resections were performed on the right side (56.1%).

### Postoperative complications

The main postoperative complication in 56.9% of the patients was an air leakage for more than 7 days. On average, chest tubes were removed between 9 and 12 days postoperatively, while the average hospitalization duration was between 10 and 13 days without a significant difference between the groups. In addition to prolonged air leakage, common complications included pneumonia (8.6%), atelectasis (10.3%), empyema (1.7%) and subcutaneous emphysema (22.4%).

Immediate postoperative mortality did not occur in our study group (0%) or during the 2-year follow-up period. Incidental PH findings occurred in 6.9% of patients, 4 patients had interstitial pneumonia, 2 patients had pulmonary aspergilloma, and 1 patient had adenocarcinoma of the lung.

Postoperative complications and hospital stays are presented in Tables 2 and 3.

**Table 2:** Postoperative complications

	Bullectomy (*n* = 19)		LVRS unilateral (*n* = 31)		LVRS bilateral (*n* = 8)		All patients (*n* = 58)	
	*n*	%	*n*	%	*n*	%	*n*	%
Reoperation	0	0	2	6.5	0	0	2	3.4
Reintubation	0	0	0	0	0	0	0	0
Mechanical ventilation	0	0	0	0	0	0	0	0
Subcutaneous emphysema	3	15.8	8	25.8	2	25.0	13	22.4
Arrhythmia	5	26.3	16	51.6	3	37.5	24	41.4
Pneumonia	3	15.8	1	3.2	1	12.5	5	8.6
Atelectasis	2	10.5	4	12.9	0	0	6	10.3
Empyema	0	0	0	0	1	12.5	1	1.7
Air leakage > 7 days	7	36.8	21	67.7	5	62.5	33	56.9
Mortality	0	0	0	0	0	0	0	0

**Table 3:** Postoperative drainage and hospital stay

	Bullectomy (*n* = 19)	LVRS unilateral (*n* = 31)	LVRS bilateral (*n* = 8)	All patients (*n* = 58)
Drain ex. postop. day, mean (SD) Min–max	9.2 [± 3.2] 5–15	10.9 [± 3.1] 6–15	12.0 [± 6.8] 7–28	10.5 [± 3.9] 5–28
Hospitalization stay, mean (SD) Min–max	10.1 [± 4.2] 5–19	12.1 [± 3.1] 6–16	13.1 [± 7.2] 8–30	11.6 [± 4.2] 5–30

Drain ex. postop day, mean = the mean amount of time until drain removal postoperatively, hospitalization stay mean = the mean amount of days spent in hospital, LVRS-unilateral = unilateral lung volume reduction surgery, LVRS-bilateral = bilateral lung volume reduction surgery

### Clinical outcome 6 months after surgery

#### Lung function and blood gas analysis

All 58 patients (100%) achieved a positive outcome: at 6 months after surgery, there was a statistically significant improvement in FEV_1_, 6MWT, mMRC, BMI and quality of life (Table 4). The mean increase in FEV_1_ was 340 ml ± 0.150 ml (*p* < 0.001), 350 ml ± 0.220 ml (*p* < 0.001) and 610 ml ± 0.270 ml (*p* < 0.001) in groups 1, 2 and 3, respectively.

**Table 4:** Clinical outcome 6 months after surgery

	Bullectomy (*n* = 19)		LVRS unilateral (*n* = 31)		LVRS bilateral (*n* = 8)	
	Mean (SD)	*p*	Mean (SD)	*p*	Mean (SD)	*p*
FEV_1_ (L)						
Pre	2.21 (0.17)	< 0.001*	2.06 (0.25)	< 0.001*	1.89 (0.20)	< 0.001*
Post	2.55 (0.15)		2.42 (0.22)		2.51 (0.27)	
TLC (L)						
Pre	5.60 (0.76)	0.004*	6.15 (0.90)	0.003*	6.51 (0.71)	0.003*
Post	4.86 (0.69)		5.46 (0.83)		5.71 (0.68)	
RV (L)						
Pre	3.39 (0.55)	0.006*	3.84 (0.66)	0.003*	4.39 (0.51)	< 0.001*
Post	2.90 (0.47)		3.32 (0.53)		3.53 (0.52)	
BMI (kg/m^2^)						
Pre	23.93 (3.23)	**0.350**	24.26 (3.50)	**0.243**	19.94 (3.86)	**0.435**
Post	24.28 (3.09)		24.38 (3.39)		19.83 (3.24)	
6MWT (m)						
Pre	395.37 (27.21)	< 0.001*	366.87 (28.37)	< 0.001*	352.50 (29.5)	< 0.001*
Post	443.16 (14.07)		431.06 (16.68)		427.62 (19.10)	
Dyspnea						
Pre	1.95 (0.23)	< 0.001*	2.06 (0.25)	< 0.001*	2.38 (0.50)	< 0.001*
Post	0.53 (0.51)		1.00 (0.00)		1.00 (0.37)	

Data expressed as mean ± SD (pre = baseline, post = 6 months postoperatively)

(L): liters; FEV_1_: forced expiratory volume; TLC: total lung capacity; RV: residual volume; BMI: body mass index; 6MWT m: 6-min walking test result in meters

Bold value indicates the statistical significance

In our analysis, no significant differences were found in between the preoperative and postoperative values related to DLCO, PaO_2_, and PaCO_2_ and LVEF.

#### Exercise capacity

There was also a statistically significant improvement in the 6-MWT with 47.8 m ± 14.07 m, 64.2 m ± 16.68 m and 75.1 m ± 19.10 m following bullectomy, unilateral and bilateral LVRS, respectively.

#### BMI

The average BMI was the only parameter that did not change significantly at 6 months after any of the compared surgeries (Table 4).

#### Symptoms and quality of life

Our analysis showed that at 6 months after surgery, there was a statistically significant decrease in the mMRC score in all the groups. There was no significant difference in the average MMRC score change across all postoperative measurement dates (Table 4).

### Clinical outcome 12 and 24 months after surgery

After 12 and 24 months, the clinical benefit decreased but most of the lung function parameters remained significantly improved compared to baseline.

Twenty-four months after bullectomy, 63.2% of our patients stated that their quality of life is improved from baseline. With regard to the use of unilateral LVRS, after 24 months, 61.3% of patients said that their quality of life is better, but this is not a significant majority compared to the number of those who thought that their quality of life is much better (38.7%, *p* = 0.209). After bilateral LVRS, the largest number of patients stated that their quality of life is better (75%), or much better (25%, *p* = 0.046), which was significantly greater than those who stated that there was no change in quality of life.

**Fig. 1:**
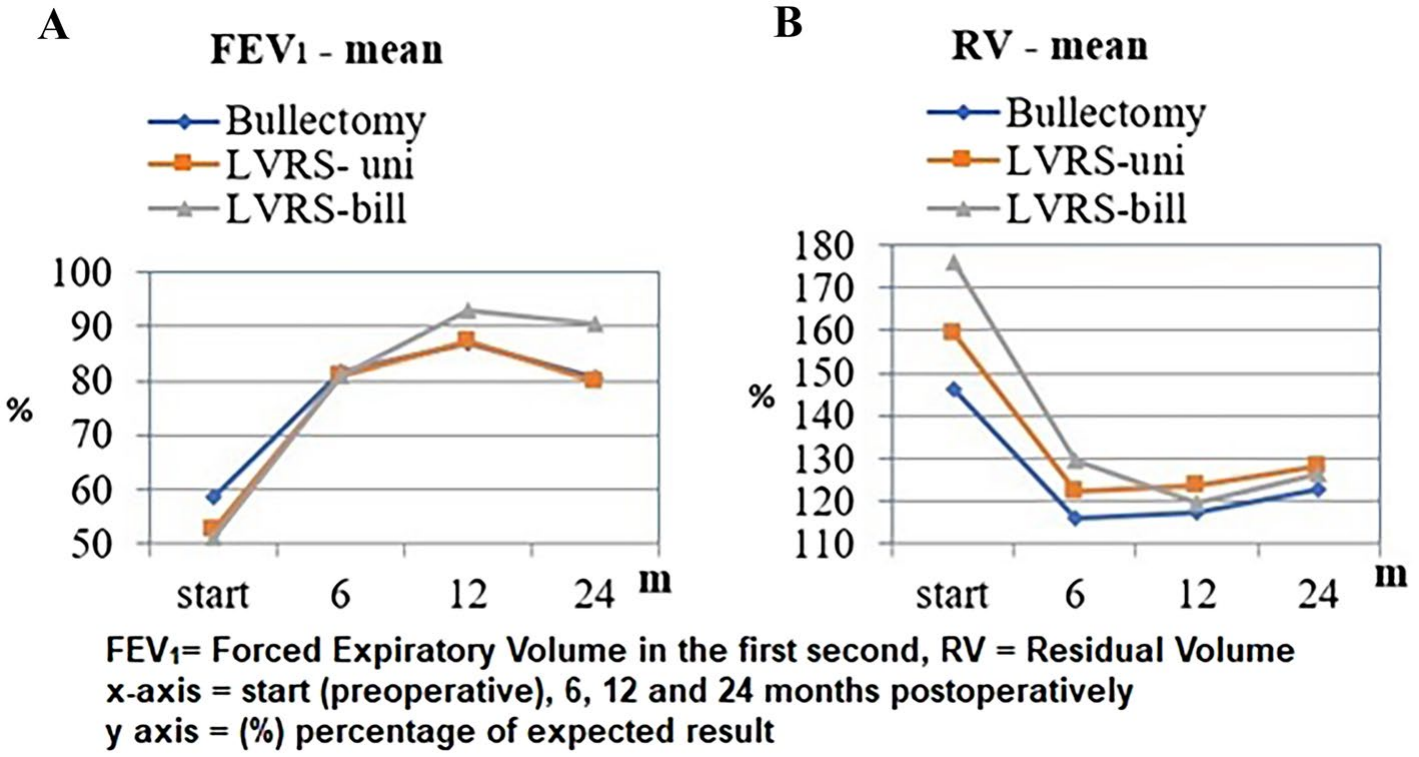
**A** FEV_1_ and **B** RV preoperative and after surgical intervention

**Fig. 2:**
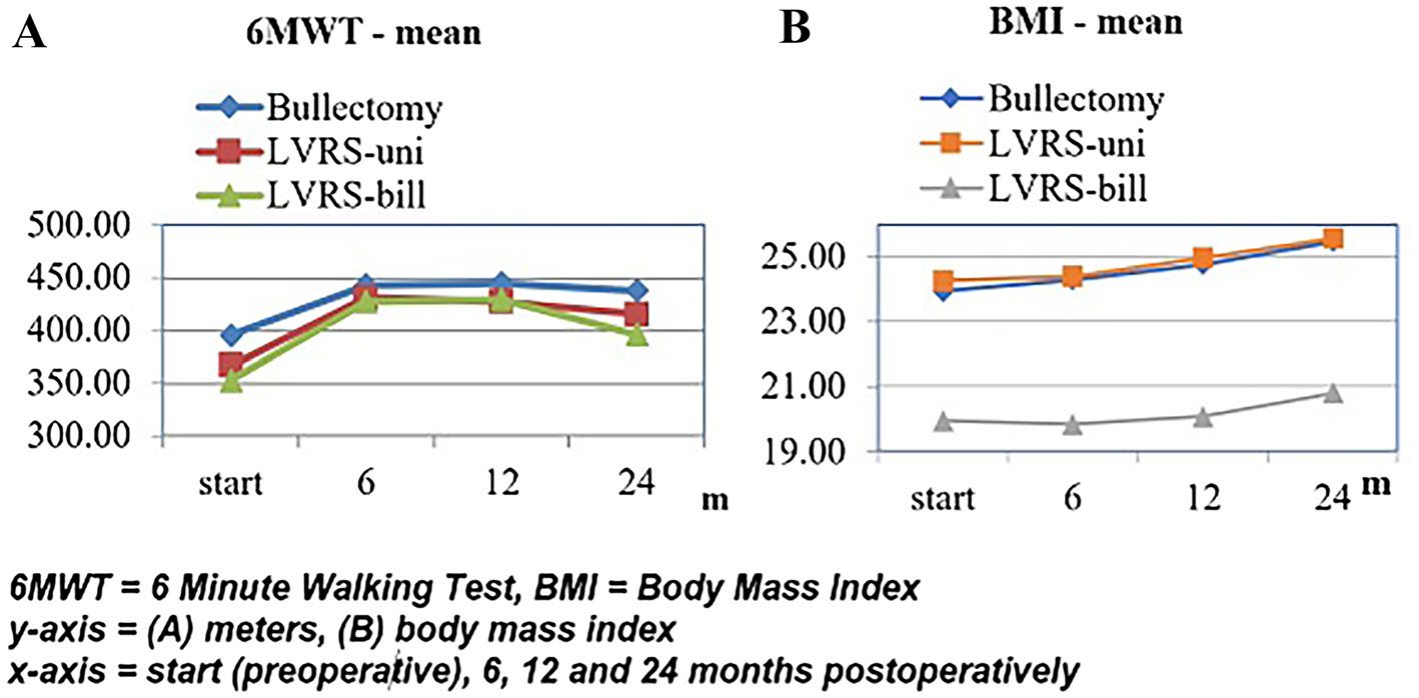
**A** 6MWT and **B** BMI preoperative and after surgical intervention

The clinical outcomes 12 and 24 months after surgery are presented in Tables 5 and 6 as well as in Figs. 1, 2.

**Table 5:** Clinical outcome 12 months after surgery

	Bullectomy (*n* = 19)		LVRS unilateral (*n* = 31)		LVRS bilateral (*n* = 16)	
	Mean (SD)	*p*	Mean (SD)	*p*	Mean (SD)	*p*
FEV_1_ (L)						
Pre	2.21 (0.17)	< 0.001*	2.06 (0.25)	< 0.001*	1.89 (0.20)	< 0.001*
Post	2.50 (0.15)		2.38 (0.22)		2.69 (0.26)	
TLC (L)						
Pre	5.60 (0.76)	0.003*	6.15 (0.90)	0.004*	6.51 (0.71)	< 0.001*
Post	4.86 (0.71)		5.49 (0.82)		5.48 (0.72)	
RV (L)						
Pre	3.39 (0.55)	0.012*	3.84 (0.66)	0.004*	4.39 (0.51)	< 0.001*
Post	2.95 (0.53)		3.35 (0.62)		3.47 (0.54)	
BMI (kg/m^2^)						
Pre	23.93 (3.23)	0.023*	24.26 (3.50)	< 0.001*	19.94 (3.86)	**0.625**
Post	24.75 (3.03)		24.97 (3.07)		20.08 (3.27)	
6MWT (m)						
Pre	395.37 (27.21)	< 0.001*	366.87 (28.37)	< 0.001*	352.50 (29.55)	< 0.001*
Post	444.79 (15.67)		427.29 (18.57)		429.38 (15.87)	
Dyspnea						
Pre	1.95 (0.23)	< 0.001*	2.06 (0.25)	< 0.001*	2.38 (0.50)	< 0.001*
Post	0.53 (0.51)		1.00 (0.00)		0.87 (0.34)	

Data expressed as mean ± SD (pre = baseline, post = 12 months postoperatively)

(L):liters; FEV_1_: forced expiratory volume; TLC: total lung capacity; RV: residual volume; BMI: body mass index; 6MWT (m): 6-min walking test in meters

Bold value indicates the statistical significance

**Table 6:** Clinical outcome 24 months after surgery

	Bullectomy (*n* = 19)		LVRS unilateral (*n* = 31)		LVRS bilateral (*n* = 8)	
	Mean (SD)	*p*	Mean (SD)	*p*	Mean (SD)	*p*
FEV_1_ (L)						
Pre	2.21 (0.17)	0.001*	2.06 (0.25)	< 0.001*	1.89 (0.20)	< 0.001*
Post	2.42 (0.17)		2.30 (0.21)		2.45 (0.22)	
TLC (L)						
Pre	5.60 (0.76)	0.005*	6.15 (0.90)	0.011*	6.51 (0.71)	0.001*
Post	4.92 (0.52)		5.57 (0.71)		5.59 (0.73)	
RV (L)						
Pre	3.40 (0.55)	**0.059**	3.84 (0.66)	0.034*	4.40 (0.51)	< 0.001*
Post	3.08 (0.53)		3.49 (0.57)		3.57 (0.54)	
BMI (kg/m^2^)						
Pre	23.93 (3.23)	0.001*	24.26 (3.50)	< 0.001*	19.94 (3.86)	**0.085**
Post	25.47 (2.83)		25.57 (2.94)		20.80 (3.00)	
6MWT (m)						
Pre	395.37 (27.21)	< 0.001*	366.87 (28.37)	< 0.001*	352.50 (29.55)	0.012*
Post	437.63 (20.25)		415.48 (16.42)		396.00 (18.83)	
Dyspnea						
Pre	1.95 (0.23)	< 0.001*	2.06 (0.25)	< 0.001*	2.38 (0.50)	< 0.001*
Post	0.53 (0.51)		0.97 (0.18)		1.00 (0.00)	

Data expressed as mean ± SD (pre = baseline, post = 24 months postoperatively)

(L): liters; FEV_1_: forced expiratory volume; TLC: total lung capacity; RV: residual volume; BMI: body mass index; 6MWT (m): 6-min walking test result in meters

Bold value indicates the statistical significance

## Discussion

COPD and emphysema, which are characterized by chronic airflow obstruction and hyperinflation, are associated with high morbidity and mortality. Conservative COPD treatment slows disease progression, but thus far, there is no curative therapeutic option. Despite optimal pharmacologic therapy and rehabilitation, most patients with advanced COPD still have symptoms that impair quality of life. In addition to medical treatment, bronchoscopic lung volume reduction, bullectomy, LVRS or lung transplantation are invasive treatment options for selected patients with advanced emphysema and can provide good long-term results.

There is still significant controversy about the role of unilateral, sequential, and bilateral lung volume reduction surgery. Many authors advocate bilateral surgery to reduce lung volume, due to greater short-term improvements in spirometry, lung volume, dyspnea, quality of life, and survival [8]. Patients with severe emphysema show clinically significant benefits after unilateral LVRS with subjective and objective improvement and denial of the need for contralateral intervention during the 3-year follow-up period [9]. The role of sequential unilateral reduction is still unclear because there is a lack of data in the literature [10].

In the present study, the NETT criteria were used for patient selection and patients with FEV_1_ ≤ 20% and DLCO ≤ 20% were excluded [6]. Patients who underwent LVRS most often had heterogeneous emphysema of the upper lobes as a result of CT findings, while in the bullectomy group, a significantly high number of patients had CT findings of bullous upper lobes in the context of emphysema (stage III according to the De Vries and Wolf classification).

Our results suggest that significant increases in lung function can be achieved by unilateral LVRS for at least 2 years and that the beneficial effect of LVRS still occurs. The results showed that all patients (100%) achieved a positive outcome. At 6-month follow-up, patients experienced a significant improvement in lung function parameters, exercise capacity and dyspnea scores. These improvements were still seen over a 24-month period, even if a gradual deterioration can be observed during this time. Thereby, patients who underwent bilateral LVRS experienced the greatest improvement but had also the greatest 1-year decrease in values in relation to the other two groups. The 1-year decrease in FEV_1_ was significant in the bilateral LVRS group (240 ml) but was not significant in the bullectomy or unilateral LVRS group (80 ml). At 6, 12 and 24 months after surgical intervention, all the subjects crossed FEV_1_ improvement ≥ 350 m, which was a significant improvement compared to the initial measurement.

Overall, it can be hypothesized that the greatest functional benefit of timing for sequential surgery may be achieved at the time of maximum improvement (i.e., approximately in the first year), to try to slow the decline in objective physiological parameters and subjective health findings with long-term benefits. Most patients were satisfied with the effects of unilateral LVRS and did not require other interventions during the observation period. This indicates that the palliative goal of the intervention can be achieved through a one-sided approach.

The most common complication after LVRS reported in many studies was a prolonged air leakage. Ciccone et al. reported that up to 45.2% of patients had prolonged air leakage after LVRS [11]. As reported in the NETT trial, surgical revision due to constant air leakage was required in up to 5% of patients [6]. In our study, 56.9% of prolonged air leaks were recorded and a decision for surgical reintervention was made in 2 patients (3.4%). Many studies have shown that the use of fibrin glue for tissue prevents air leakage during lung resection, especially after LVRS [12]. In addition, prolonged postoperative air leakage has been associated with prolonged hospital stays, increased hospital costs, and an increased incidence of cardiopulmonary complications [13]. Fibrin tissue adhesives were used in all 58 patients to prevent prolonged air leakage. On average, chest tubes were removed between the 9th to the 12th postoperative days. The length of hospitalization ranged from an average of 10 to 13 days; in extreme cases, bullectomy chest tube removal occurred at a minimum of 5 days, and in bilateral LVRS it occurred at a maximum of 30 days.

Pneumonia is considered to be the second most common pulmonary complication after LVRS, in the NETT trial approximately 18% of patients developed pneumonia postoperatively [6]. In our analysis, 8.6% of patients developed pneumonia. To minimize infectious complications, prophylactic antibiotic therapy began in our group one hour preoperatively and continued until chest tubes were removed. Also, the number of cardiac complications such as arrhythmia, myocardial infarction, and pulmonary embolism after LVRS are similar to those after other thoracic surgeries [14]. In our study, arrhythmia was the most common cardiac complication after LVRS (41.4%) and only required medical cardioversion.

In our study, coexisting lung cancer was detected in one patient (1.72%). Patients who are candidates for LVRS could be potential candidates for careful evaluation of possible lung cancer for which the detection rate is 1–4% [15, 16].

In the NETT trial, the mortality rate was 5.5% 90 days after surgery, and 24% of those who underwent unilateral LVRS produced similar improvements in lung function compared to bilateral LVRS with a lower mortality and morbidity and shorter hospital stays [6].

This research paper did not identify the baseline factors that predict patients who achieved the best improvements compared with those who achieved fewer improvements after surgical intervention. Possible limitations of this study were that we had a relatively small patient group; all of our patients were active smokers or former smokers and we had a large proportion of male patients overall. Unfortunately, due to institutional constraints in terms of equipment, material resources and surgical competency during the time period retrospectively analyzed, VATS was available for only a small, specific group of patients, and only patients who underwent muscle-sparing thoracotomy were included in this series to avoid potential bias.

Unfortunately, due to institutional constraints in terms of equipment, material resources and surgical competency during the time period retrospectively analyzed VATS was only available for a small, specific group of patients and only patients who underwent a muscle-sparing thoracotomy were included in this series to avoid potential bias.

## Conclusion

During our 2-year follow-up period, bullectomy and unilateral LVRS had results comparable to those of sequential bilateral LVRS in terms of lung function, health, recovery time, and reduced morbidity. However, controversy exists over whether the results are better after unilateral or bilateral LVRS on long-term monitoring [17–19].

The results of a successful surgical treatment of pulmonary emphysema do not depend only on good surgical technique and postoperative care. Proper selection and a multidisciplinary approach for patients with emphysema contribute to positive outcomes. Since the longevity of functional improvement provided by LVRS has not yet been determined, unilateral intervention with the option of subsequent reduction, i.e., contralateral volume reduction, may serve to prolong the palliative benefits of this therapy.

## Data Availability

To request a more extensive dataset, please email the corresponding author.
